# Structure-Activity Relationship of Chlorotoxin-Like Peptides

**DOI:** 10.3390/toxins8020036

**Published:** 2016-02-02

**Authors:** Syed Abid Ali, Mehtab Alam, Atiya Abbasi, Eivind A. B. Undheim, Bryan Grieg Fry, Hubert Kalbacher, Wolfgang Voelter

**Affiliations:** 1 International Center for Chemical and Biochemical Sciences (ICCBS), HEJ Research Institute of Chemistry, University of Karachi, Karachi-75270, Pakistan; promet213@yahoo.com (M.A.); atiya786@super.net.pk (A.A.); 2 Interfaculty Institute of Biochemistry (IIB), University of Tuebingen, Hoppe-Seyler Str. 4, Tuebingen D-72076, Germany; kalbacher@uni-tuebingen.de (H.K.); Wolfgang.Voelter@uni-tuebingen.de (W.W.); 3 Venom Evolution Laboratory, School of Biological Sciences, University of Queensland, Brisbane, QLD 4072, Australia; e.undheim@uq.edu.au (E.A.B.U.); bgfry@uq.edu.au (B.G.F.)

**Keywords:** CFTR, chlorotoxin, chloride channel, MMP2, peptidyl-inhibitors, scorpion venom

## Abstract

Animal venom (e.g., scorpion) is a rich source of various protein and peptide toxins with diverse physio-/pharmaco-logical activities, which generally exert their action via target-specific modulation of different ion channel functions. Scorpion venoms are among the most widely-known source of peptidyl neurotoxins used for callipering different ion channels, such as; Na^+^, K^+^, Ca^+^, Cl^−^, *etc.* A new peptide of the chlorotoxin family (*i.e.*, Bs-Tx7) has been isolated, sequenced and synthesized from scorpion *Buthus sindicus* (family *Buthidae*) venom. This peptide demonstrates 66% with chlorotoxin (ClTx) and 82% with CFTR channel inhibitor (GaTx1) sequence identities reported from *Leiurus quinquestriatus hebraeus* venom. The toxin has a molecular mass of 3821 Da and possesses four intra-chain disulphide bonds. Amino acid sequence analysis of Bs-Tx7 revealed the presence of a scissile peptide bond (*i.e.*, Gly-Ile) for human MMP2, whose activity is increased in the case of tumour malignancy. The effect of hMMP2 on Bs-Tx7, or *vice versa*, observed using the FRET peptide substrate with methoxycoumarin (Mca)/dinitrophenyl (Dnp) as fluorophore/quencher, designed and synthesized to obtain the lowest *K*m value for this substrate, showed approximately a 60% increase in the activity of hMMP2 upon incubation of Bs-Tx7 with the enzyme at a micromolar concentration (4 µM), indicating the importance of this toxin in diseases associated with decreased MMP2 activity.

## 1. Introduction

Scorpions, the most ancient arthropods on Earth, can be divided phylogenetically into 18 distinct families consisting of more than 1500 species. Scorpions (Phylum *Arthropoda*, Class *Arachnida*, Order *Scorpionida*) possess a venom apparatus made up of a vesicle holding a pair of venom glands connected to the telson, which is used for injecting the venom. At least thirty species have been identified as potentially toxic to humans [1]. The main venomous families are *Buthidae* (the largest family with 82 genera and 773 species) and *Chactidae* (12 genera and 152 species). The venom produced by these scorpions is a complex cocktail of protein and peptide toxins targeting ion channel functions of excitable and non-excitable cells [2,3]. The most remarkable feature is the presence of a large number of closely-related toxins (*i.e.*, natural combinatorial libraries) in the venom, suggesting recent gene duplication events [4].

Classification of these toxins is important for understanding the structure-function relationship of each individual group. The major criteria used for classification are based on receptor/ion channel specificity (e.g., K^+^, Na^+^, Ca^2+^ and Cl^−^), peptide length (e.g., short- and long-chain), structural scaffold (α, αβ and βαββ), disulfide bonds (3 or 4 and pairing pattern), the mechanism of action/binding sites (α- or β-like toxins), their cellular target, *etc.* [2,3,5]. Thus, at least 12 subfamilies of K^+^-channel-specific toxins have been identified based on sequence similarity [6]. Similarly, more than 200 scorpion toxins specific for K^+^, Na^+^, Ca^2+^ and Cl^−^ channels have been reported so far. These toxins have been grouped into 14 subfamilies of K^+^ and Cl^−^ channel toxins and 12 subfamilies of Na^+^ and Ca^2+^ toxins. The scorpion long-chain toxins comprise 60–70 amino acids and target Na^+^ channels effectively, whereas the short-chain toxins (containing 20–40 amino acids) recognize mainly K^+^ and Cl^−^ channels [2,3]. In addition, some scorpion toxins with variable peptide lengths have been found to modulate the Ca^2+^ channel and are placed in a separate structural group [7]. Interestingly, a new structural group possessing two conserved structural motifs in K^+^ and/or Na^+^ channels for scorpion toxins has also been reported [8,9]. Attempts have also been made to classify scorpion toxins according to the species targeted (*i.e.*, anti-insect, arthropod, crustacean and/or mammal), although some toxins exhibit cross-specificity [10]. 

The scorpion short-chain toxins (namely chlorotoxin and chlorotoxin-like peptides) have been found to influence Cl^−^ channel activity. A number of studies on chlorotoxin [11] and chlorotoxin-like peptides, such as ClTx-a,b,c,d, BmKCL1, Lqh-8:6, Be I5A, BeI1, AmmP2 and GaTx1 [12,13,14], reveal interesting characteristics. For example, chlorotoxin, found in the venom of the death-stalker scorpion (*Leiurus quinquestriatus hebraeus*), is a small, basic 36-amino acid residue peptide stabilized by four disulfide bonds. It is a high affinity ligand, which blocks the small conductance chloride channels and, thus, was initially used as a pharmacological tool to characterize chloride channels [11]. In contrast, despite its close sequence analogy with chlorotoxin (*i.e.*, 75%), GaTx1, reported from the same scorpion venom, proved to be a highly-specific blocker for the CFTR channel. On the other hand, studying glioma-specific chloride current, it was discovered that chlorotoxin also possesses targeting properties towards cancer cells, including glioma, melanoma, small cell lung carcinoma, neuroblastoma and medulloblastoma [14]. Despite the un-established mode of action and/or cell surface targets, this class of peptides has shown great clinical potential. For example, the synthetic version of chlorotoxin is in phase III clinical trial, while the ^131^I-labeled synthetic member (namely 131I-TM-601) was already approved by the FDA and in use for the treatment of malignant gliomas [15]. Moreover, many chlorotoxin-based conjugates have also been developed for effective molecular imaging and targeting therapies [16]. These conjugates not only have the capability to pass the blood brain barrier (BBB) and selectively bind to the cell surface of glioma, but also have the ability to exert imaging and therapeutic functionalities. For example, fluorescently-labelled chlorotoxin (*i.e.*, CTX:Cy5.5) was proven to be the most sensitive probe for intraoperative visualization of cancer foci and found to be approximately 500-times more sensitive than normal MRI [17]. Likewise, the multifunctional chlorotoxin-conjugated magnetic nanoparticles, which are fluorescently-labelled, can be used for both MR/fluorescence imaging of glioma, as well as drug (e.g., DOX) and gene (e.g., siRNA) delivery systems for targeted chemo and gene therapies, respectively [14,16].

In continuation of our earlier studies, we have isolated a new chlorotoxin-like peptide (Bs-Tx7) from the venom of a common yellow scorpion, *Buthus sindicus* (family *Buthidae*), from the Sindh province of Pakistan. The isolated peptide has been sequenced, as well as synthesized. The isolated toxin demonstrates 66% with chlorotoxin (ClTx) and 82% with the CFTR channel inhibitor (GaTx1) sequence identities reported from *Leiurus quinquestriatus hebraeus* venom. Amino acid sequence analysis of Bs-Tx7 revealed the presence of a scissile peptide bond (*i.e.*, Gly-Ile) for human MMP2, a potential marker of cellular tumours. Thus, in order to differentiate, Bs-Tx7 and chlorotoxin have been subjected to an hMMP2 enzyme inhibition assay using an FRET peptide substrate, suggesting a further functional bifurcation within the class of chlorotoxins and chlorotoxin-like peptides. 

## 2. Results

### 2.1. Purification and Structural Elucidation of Peptide Bs-Tx7

Purification of scorpion *Buthus sindicus* venom components was performed using single-step RP-HPLC, as described in the Experimental Section. Approximately 41 peaks were collected manually. Primary structure elucidation studies on Peaks 1, 5, 6, 8, 10 and 14 have been reported earlier and identified as scorpion venom short-chain neurotoxins [13,18]. This report describes purification, primary structure elucidation, synthesis and complete characterization of another minor Peak 7, designated as Bs-Tx7 (Figure S1). The measured monoisotopic MH^+1^ mass of the main component of Peak 7 was 3821.46 Da.

**Figure 1:**
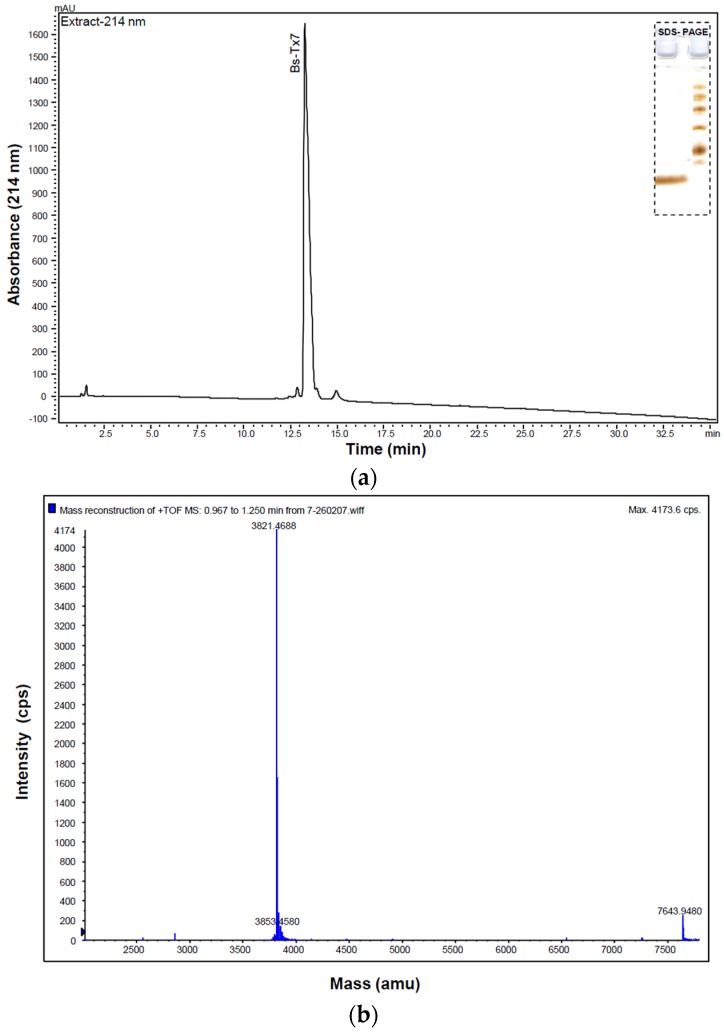
(**a**) Final purification of Bs-Tx7 (retention time 36.2 min; from Figure S1) was achieved by re-chromatography on a C2/C18 (3 µ, 100 × 4.6 mm) RP-FPLC column using a linear gradient program as follows: Eluent A, 0.1% trifluoroacetic acid in water; Eluent B, 100% acetonitrile containing 0.05% TFA; gradient program, 0%–40% B for 50 min at a flow rate of 1 mL/min. The eluate was monitored at 214 nm. Insert: 15% SDS PAGE analysis of the toxin Bs-Tx7 (left Lane 1) and standard molecular weight markers (right Lane 2); (**b**) Deconvoluted ESI-MS spectrum of the purified Bs-Tx7 determined on QStar XL MS using positive ion mode giving the monoisotopic mass of 3821.46 Da. A good agreement between the mass calculated from the established sequence and mass measured by MS has been observed.

**Figure 2:**
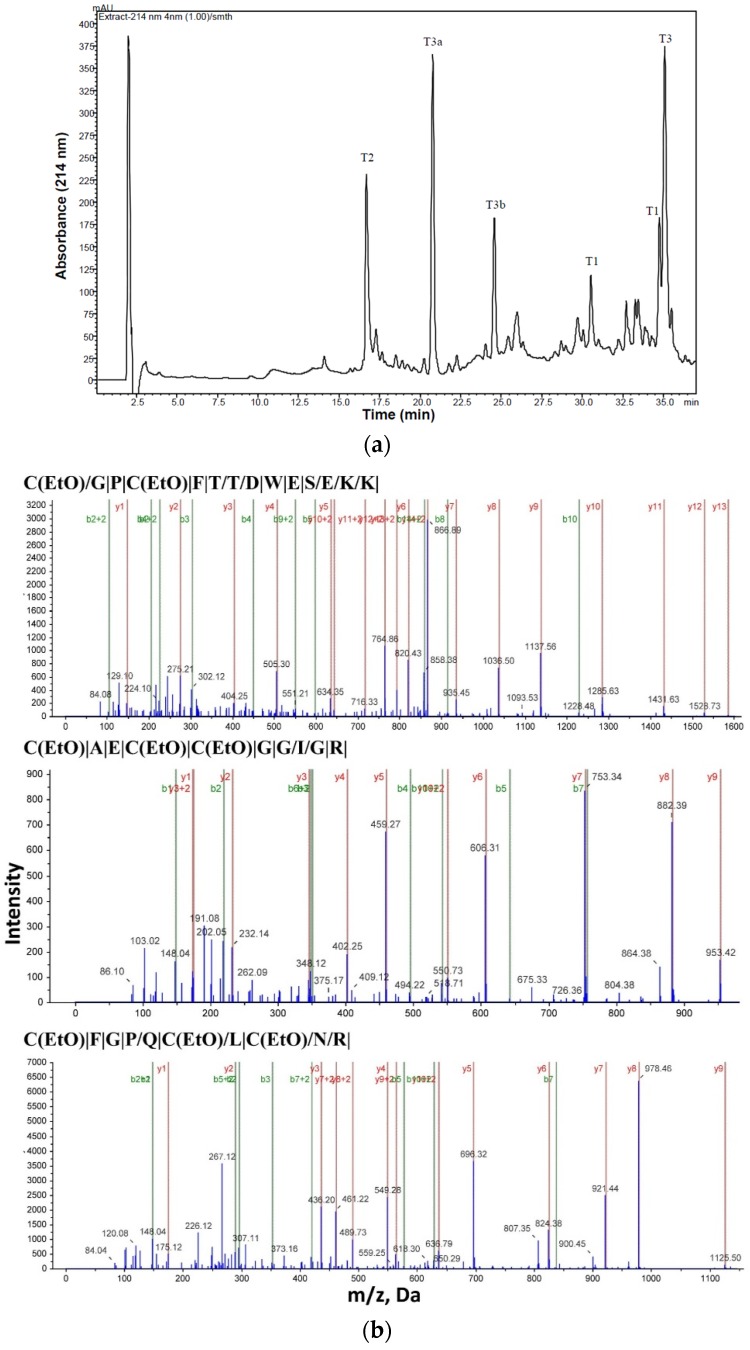
(**a**) Separation profile of the tryptic digest of Bs-Tx7 on the C12 RP-HPLC column (4 µ, 100 × 4.6 mm) using a linear gradient program as follows: Eluent A, 0.1% trifluoroacetic acid in water; Eluent B, 100% acetonitrile containing 0.05% TFA; gradient program, 0%–40% B for 50 min at a flow rate of 1 mL/min. The peaks were collected manually, vacuum dried and subjected to mass and Edman sequencing. T stands for tryptic digest; variables “a” and “b” represent nonspecific cleavage products (see Table 1); (**b**) Representative shotgun-derived [19] MS2 spectra and corresponding sequences of tryptic fragments providing 97% coverage of the mature sequence of Bs-Tx7. Fragment types are indicated in each spectrum and shown in the corresponding sequence as singly- or doubly-charged b-ions (\), *y*-ions (/) or both (|).

The minor fraction collected as Bs-Tx7 (Figure S1; [18]) was re-chromatographed on a C2/C18 column (Figure 1a). The purity of the peptide Bs-Tx7 was confirmed by 15% SDS PAGE gel (insert of Figure 1a) and MALDI-TOF MS (Figure 1b). The primary structure of the purified Bs-Tx7 was established by automated gas phase sequencing (yield >95%). Pairing of disulfide bonds in the native peptide was determined after tryptic cleavage for 24 h at 37 °C. Off-line separation of the tryptic digest was performed using RP-C12-HPLC (Figure 2a). All peaks were subject to a purity check by mass spectrometry, followed by Edman sequencing (Table 1). Trypsin-digested, reduced and alkylated Bs-Tx7 was also analysed by LC-ESI-MS/MS (Figure 2b), which further yielded the complete primary structure. The calculated mass of the obtained primary sequence of Bs-Tx7 was in good agreement with that observed, assuming all eight cysteines form disulfide bonds. The positions of these disulfide bonds were confirmed using established techniques [13,18] and additionally by the solid phase peptide synthesis.

**Table 1:** Amino acid sequence and ESI-MS of peptides obtained from tryptic cleavage of oxidized Bs-Tx7 (peptides obtained from Figure 2a).

Peptide	Residue Number	Sequence *	Mass Observed	Mass Calculated	Missed Cleavage
***Tryptic cleavage products-Oxidized:***					
T1	1–14	CGPCFTTDWESEKK	1741.8307	1741.8578	1
T2	15–24	CAECCGGIGR	1112.1258	1113.1495	-
T3	25–35	CFGPQCLCNRK	1413.6722	1413.556	1
***Non specific cleavage products:***					
T3a	25–29	CFGPQ	599.2600	599.17	0
T3b	30–35	CLCNRK(2K^+^, H^+^)	816.3808	814.116	0

* Sequences established by automated gas phase Edman degradation analysis.

### 2.2. Synthesis and Folding of Bs-Tx7

**Figure 3:**
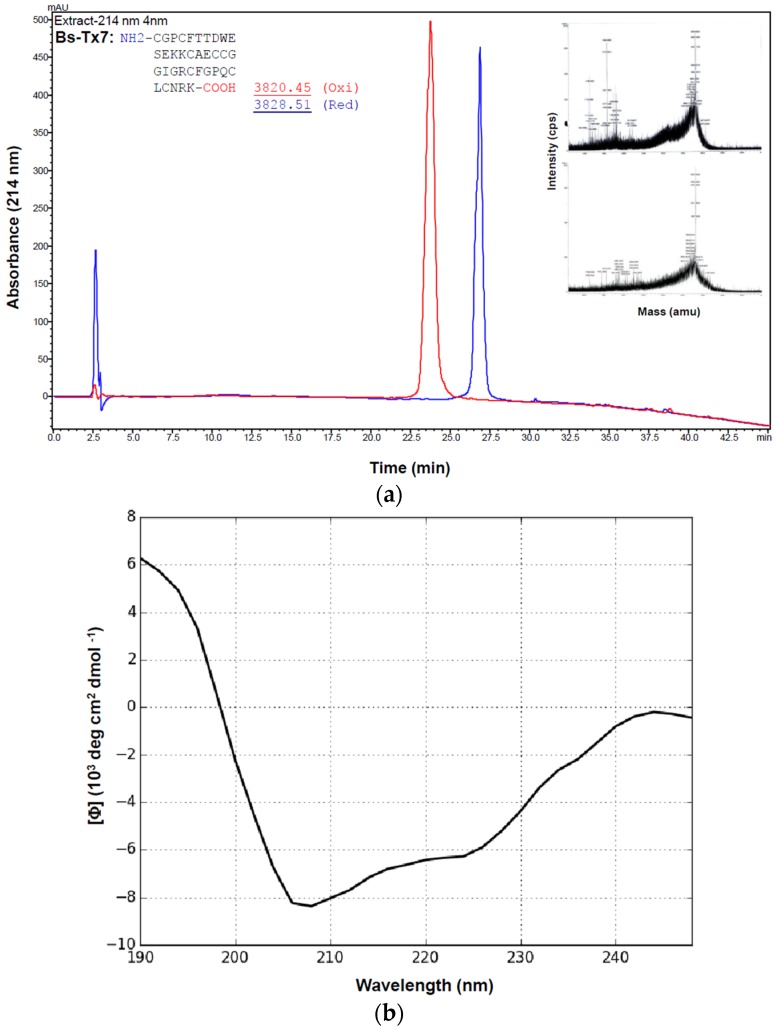
(**a**) Solid-phase peptide synthesis of Bs-Tx7 using a Syro-II peptide synthesizer and oxidized as recently described by us [20,21,22]. Superimposed are the chromatograms of the purified synthetic Bs-Tx7 in reduced (blue) and oxidized (red) forms. The sample was loaded on an RP-HPLC column (C2/C18) and manually collected using a gradient program as described in Figure 1a. Insert: the average molecular masses of the collected peptides determined by MALDI-TOF MS; (**b**) Far-UV (190–250 nm) CD spectrum of the synthetic Bs-Tx7 (~0.1 mg/mL) performed in 10 mM phosphate buffer, pH 7.4, collected with a J810 Jasco spectropolarimeter at 0.5-nm resolution using a sample cell of a 1-mm path length [21].

Bs-Tx7 was synthesized and folded as described previously (Figure 3a) [20]. The average molecular masses of the toxins obtained in reduced and oxidized form show a difference of 8 Da, indicating the presence of eight cysteine residues and the formation of four disulfide linkages in the native peptide. Proper folding of Bs-Tx7 to its native form was further complemented by CD analysis (Figure 3b), as well as confirmation that the native and synthetic peptide exhibit the same activity (see the following sections), identical mass and retention time. Moreover, chlorotoxin as the positive control [20], as well as Bs-Tx8 (Figure S2), as a close sequence homologue (80%) of *B. sindicus* venom [13], were also synthesized for comparison.

### 2.3. In Silico Studies on Bs-Tx7

**Figure 4:**
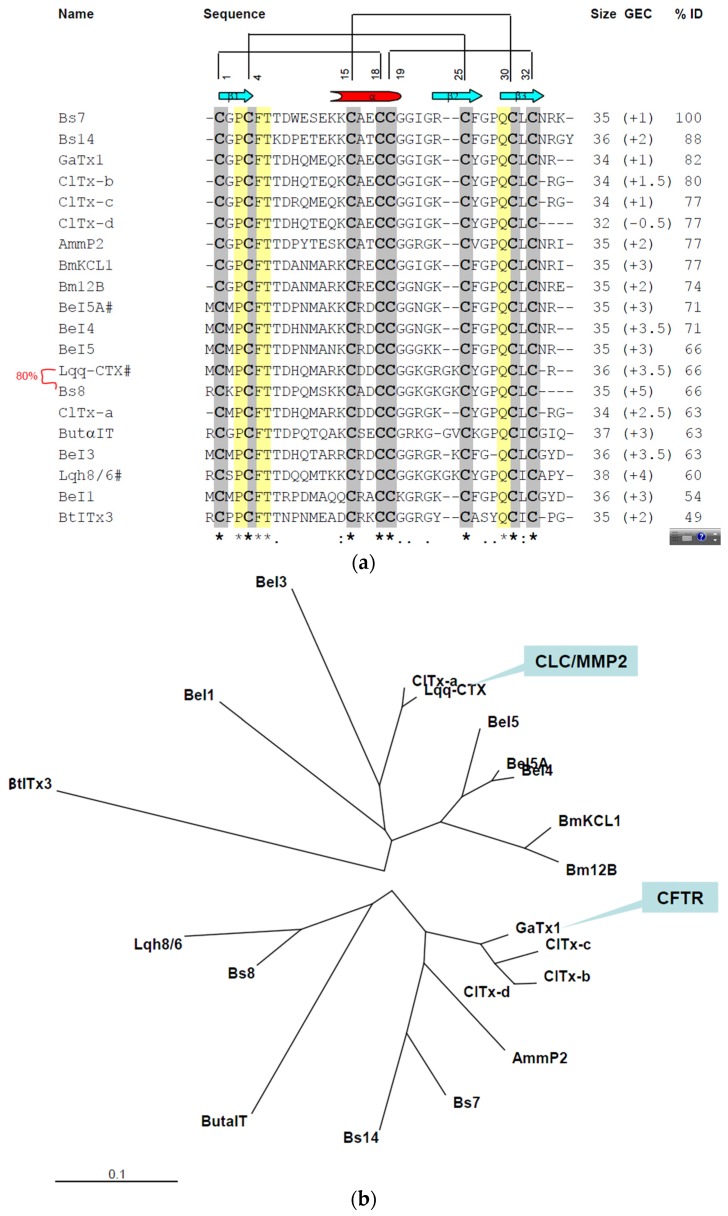
(**a**) Multiple sequence alignment of the Bs-Tx7 with other closely-related chlorotoxin and chlorotoxin-like peptides. Sequences were obtained from SWISS-PROT and/or the PDB database using the program FASTA/BLAST [23,24,25] and were aligned according to their conserved cysteine residues (bold letters) and their known 3D structures (marked by #) using the ClustalXv1.83 software [26]. Consensus sequences are marked by an asterisk, secondary structural elements by a cylinder and arrow (helix and sheets, respectively) and possible pairing of the disulfide linkages by lines at the top; (**b**) Phylogenetic tree of Bs-Tx7 and its close homologues derived from their primary sequences using the neighbour-joining distances method within the Phylip package [27].

Multiple sequence alignment of Bs-Tx7 was performed with its homologues obtained from BLAST searches and the literature review. Bs-Tx7 shares variable (49%–88%) sequence identity with other chlorotoxin-like, CFTR inhibitor-like and/or insectotoxin-like peptides. Bs-Tx7 revealed the highest sequence identity (88%) with Bs-Tx14 peptide previously sequenced by us, but so far functionally uncharacterized [13], and 82% identity with CFTR inhibitor “GaTx1” toxin, isolated from scorpion *L. quinquestriatus hebraeus.* Unfortunately, 3D structural detail of “GaTx1” is not yet established, but the structures of some other members, such as chlorotoxin (66%) and insectotoxins Lqh8/6 (60%) and BeI5A (71%) have been resolved by NMR spectroscopy. The 3D structural models of Bs-Tx7 and GaTx1 have been established using known coordinates and rigorously utilized for structural comparisons (Figure 4a,b). 

**Figure 5:**
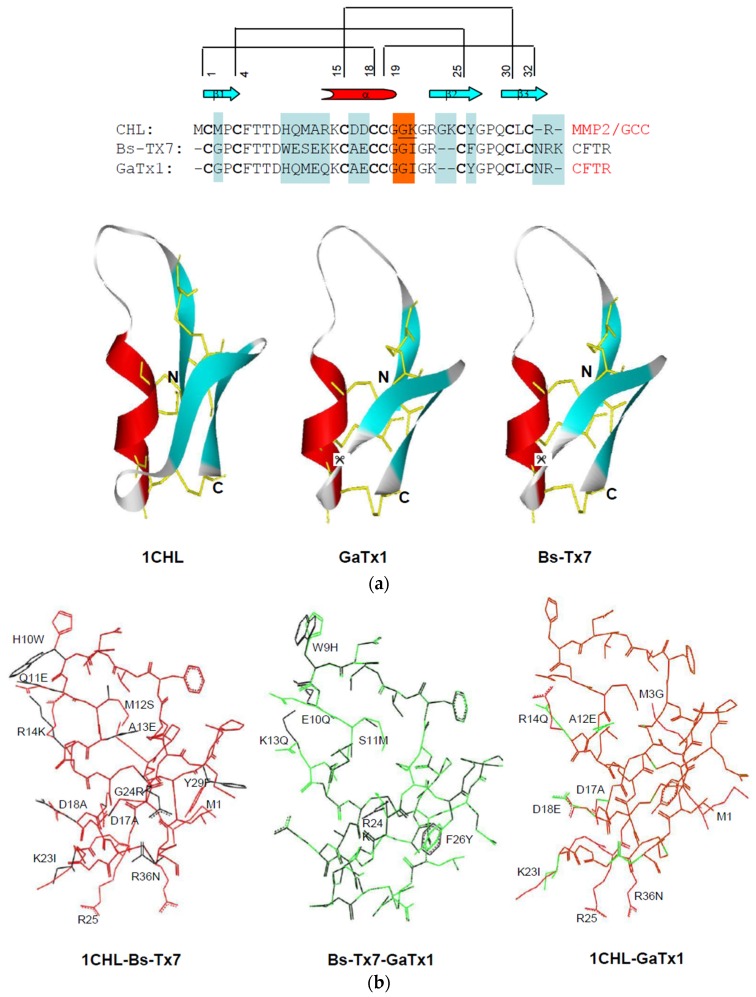

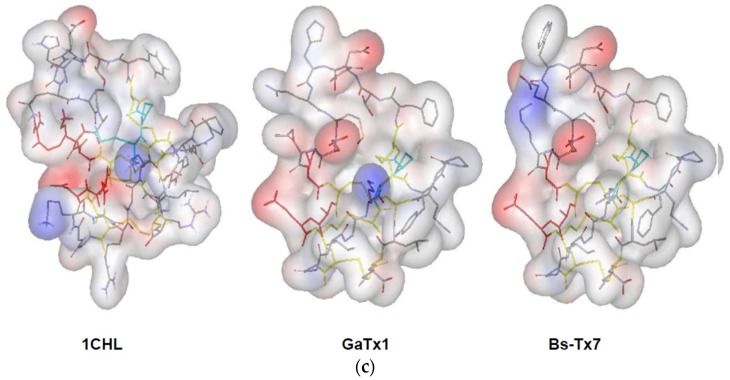
(**a**) Primary and three-dimensional structural comparison of Bs-Tx7 with chlorotoxin (MMP2/GCC inhibitor) and chlorotoxin-like peptide GaTx1 (CFTR-channel inhibitor) revealed a highly-conserved typical structural scaffold, *i.e.*, an α-helix (red), three-antiparallel β-sheets (cyan) connected by variable loops (silver) and stabilized by four intra-chain disulfide linkages (shown in yellow sticks). The known structure of chlorotoxin (PDB i.d. 1CHL) was obtained from the Protein Data Bank [28], while coordinates of Bs-Tx7 and GaTx1 were established by protein homology modelling [29,30]; (**b**) Structural superposition of the Cα-traces of Bs-Tx7 with chlorotoxin and GaTx1; colour illustrations, including chlorotoxin (red), Bs-Tx7 (black) and GaTx1 (green), revealed that structural heterogeneity and side chain conformational variation exist; (**c**) Electrostatic surface potential of Bs-Tx7 shown in comparison with chlorotoxin and chlorotoxin-like peptide GaTx1. The colour codes represent acidic (red), basic (blue) and neutral (white). All molecules are shown in identical orientation. The presence of scissile peptide bond (*i.e.*, Gly-Ile) for hMMP2 in the primary structure is highlighted and residues underlined, while in 3D structures by a sign of scissor.

Comparison of the three-dimensional homology model structure of Bs-Tx7 and GaTx1 revealed a typical and conserved structural scaffold or motif common to all scorpion short-chain neurotoxins (Figure 5a), *i.e.*, a short α-helix, two or three-antiparallel β-sheets connected by variable loops. However, in contrast to the α-KTx3 family, these are stabilized by four intra-chain disulfide linkages. Structural superposition of Bs-Tx7 with known chlorotoxin (Cltx/CHL) and modelled CFTR channel inhibitor ‘GaTx1’ shows a root mean square (RMS) deviation of less than 1 Å. Despite high sequence identity and conserved 3D structures among the three members of the MMP2/GCC/CFTR inhibitor family, subtle differences in terms of residual change, side chain conformations (Figure 5b), overall surface complementarities and molecular surface electrostatic charge distribution (Figure 5c) have been observed and seem crucial in channel blocking activity and selectivity.

### 2.4. MMP2 Activation by Bs-Tx7

**Figure 6:**
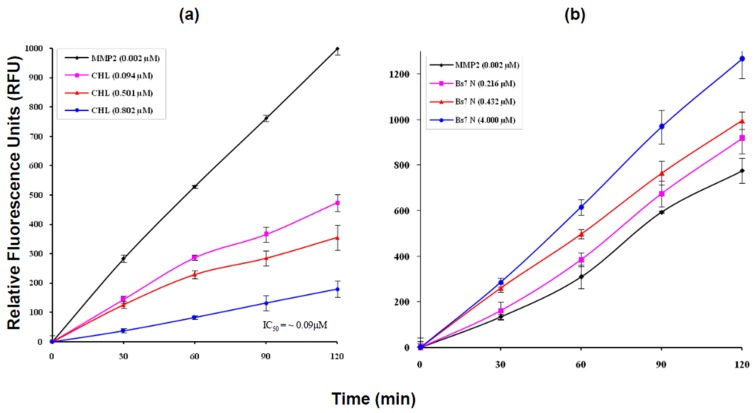
Time progress curves of hMMP2 showing the hydrolysis of the internally-quenched synthetic fluorescent substrate [20] without (0.002 µM hMMP2 alone as the control) and with different concentrations of the synthetic chlorotoxin inhibitor (**a**) and BsTx7 (**b**). Experimental data represent the mean ± SE of three experiments.

**Figure 7:**
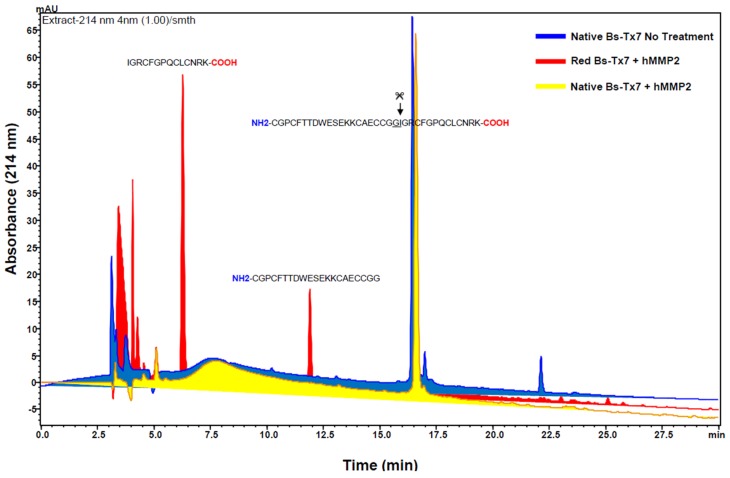
Comparative separation profiles of the Bs-Tx7 (native untreated as the control) and native and reduced synthetic Bs-Tx7 (test) treated with hMMP2 as the substrate under identical reaction conditions as described in Figure 6. RP-HPLC separation was performed on a C12 column using a gradient program as described in Figure 2a. The peaks were manually collected, vacuum-dried and subjected for Edman sequencing for *N*-terminal identification.

Bs-Tx7, tentatively identified as a CFTR inhibitor-like peptide on the basis of close sequence homology with “GaTx1” from *L. quinquestriatus hebraeus* (82%), was also tested for hMMP2 inhibiting activity with the anticipation that probably the whole family of chlorotoxin-like peptides possesses such activities. In contrast to chlorotoxin (Figure 6a [20]) and other peptides, e.g., Bs-Tx8 (having sequence homology of 80% with Cltx), which inhibits hMMP2 activities (Figure S3), different concentrations of Bs-Tx7 were found to increase the activity of hMMP2 under identical assay conditions (Figure 6b). A close sequence and structural comparison (Figure 5a) revealed the presence of a scissile peptide bond (*i.e.*, Gly-Ile) for human MMP2, whose activity is increased in the case of tumour malignancy. Interestingly, this scissile peptide bond (Gly-Ile), which resides in the loop region between the α-helix and β2-strand, was not present in chlorotoxin and other chlorotoxin-like peptides and seems to be characteristic for CFTR inhibitor “GaTx1” and GaTx1-like peptides, such as Bs-Tx7 (Figure 4a,b); suggesting the possibility for further bifurcation in the chlorotoxin-like peptide family into true chlorotoxins (which bind with the previously-proposed annexin-2/hMMP2/CLC3 complex and inhibit hMMP2 activity) and CFTR-inhibitor peptides. In order to complement this observation, the effect of hMMP2 on Bs-Tx7 (both, reduced and oxidized forms) or *vice versa* was also observed by RP-HPLC analysis of the reaction mix (Figure 7). The results obtained nicely complement our aforementioned observation that linear Bs-Tx7 was indeed cleaved into two peaks, and *N*-terminal sequencing of each generated peptide confirmed the position of the scissile peptide bond (*i.e.*, Gly-Ile). While as compared to the control (*i.e.*, native Bs-Tx7 without hMMP2 treatment), native Bs-Tx7 treated with hMMP2 eluted as such with a minor shift in retention time indicative for the nick produced by hMMP2 (which opens up the fold and increases the hydrophobicity), the cleaved peptide remained linked due to the disulfide bonds (Figure 7; Figure 5a).

## 3. Discussion

Scorpions are spread throughout the world, with the exception of Antarctica, surviving in a wide variety of habitats [31]. Primary structures of some major peptides from Pakistani common yellow scorpion *B. sindicus* have already been reported by us [13,18,32] and the functional characteristics evaluated. This study describes isolation and structural elucidation of another minor peptide ‘Bs-Tx7’ established in combination with Edman sequencing and state-of-the-art mass spectrometric techniques (Figure 1 and Figure 2, Table 1). The newly isolated peptide ‘Bs-Tx7’ has also been synthesized for complete characterization and structure-activity relationship studies (Figure 3). Sequence analysis of Bs-Tx7 shows the highest sequence identity (82%) with a known characterized CFTR inhibitor ‘GaTx1’ (Figure 4). Both toxins share also the same electrostatic charge (*i.e.*, +1), but show subtle differences in side chain conformation, as observed from 3D backbone superposition and electrostatic surface potentials (Figure 5). Beside conserved cysteine residues and the ‘CCGG’ sequence motif, some of the peptides (such as Bs-Tx7, Bs-Tx14, GaTx1 ClTx-a,b,c,d and BmKCL1) have scissile peptide bonds (*i.e.*, Gly-Ile) in the sequence for MMP2, and the increase in the FRET activity of MMP2 in the presence of Bs-Tx7 provides convincing evidence that Bs-Tx7 is a natural substrate for MMP2 (Figure 6 and Figure 7). To the best of our knowledge, this is the first report about the presence of an MMP2 natural substrate in scorpion venom. This finding may also help in resolving the issue of, *i.e.*, the inhibition of chloride and the CFTR channel by chlorotoxin and GaTX1 when applied from the cytoplasmic side, respectively. It is well known that MMP2 is expressed predominantly in extracellular matrix and, thus, catalyses the scissile peptide bond in CFTR inhibitor/-like peptides. This is one reason why a change in current has not been observed by several studies upon extracellular application of the toxin. MMP2 is also present, mostly in the active form, at the cytoplasmic site of the plasma membrane [33]. Chlorotoxin or chlorotoxin-like peptides act as MMP2 inhibitors mainly by interacting with MMP2 on the extracellular side of the membrane, resulting not only in inhibition of MMP2 activity, but also the internalization of the MMP2 co-localized glioma-specific chloride channel (GCC) responsible for the change in chloride-specific current and anti-metastasis or antitumor effect without affecting normal cells [15,33]. 

The presence of venom peptidyl libraries with properties like signaling, regulation (both excitatory and inhibitory), transport, *etc.*, is undoubtedly a unique natural gift, which has found use in the treatment of a number of life-threatening diseases. Studies on scorpion *L. quinquestriatus quinquestriatus* venom led to the identification of a specific small conductance epithelial chloride channel blocker chlorotoxin (4070 Da) [11]. Since then, many reports have appeared the use of chlorotoxin for analysing various chloride channels. However, insights into the structure and function of anion channel have strongly grown during the last decade [14,16]. Characterization of the first anion channel family and the discovery of diseases resulting from anion channel defects have been major events [34]. Soroceanu and co-workers [35] used chlorotoxin primarily for targeting brain tumours and showed by radio receptor SDS-PAGE analysis that chlorotoxin binds with a 72-kDa protein, although its identity as a glioma-specific chloride channel (GCC) or GCC-modulating receptor could not be established. Part of the ambiguity was resolved after physiological experiments, which showed that chlorotoxin does not inhibit volume-regulated, calcium-activated, cyclic AMP-activated (CFTR) chloride channels and GCC at all [36].

A review of the literature shows that only a few chloride channel blockers or chlorotoxin-like peptides have been identified from scorpions or other fauna during the last two decades [11,14]. Furthermore, the ambiguities raised by Debin’s work have now been cleared by the identification of MMP2 as the direct target of chlorotoxin [37] and the glioma-specific chloride channel possibly as the CLC3 family of chloride channels and both targets co-localized and over-expressed in tumour cells. Unlike the other scorpion toxins, chlorotoxin does not bind to a chloride channel directly; instead, it binds with MMP2 and has been explored as a primary receptor site for chlorotoxin on the surface of glioma cells. The binding of chlorotoxin with MMP2 and MTI-MMP complex causes internalization of this complex along with CLC3 channels into caveolar rafts, which depletes the available membrane-associated chloride channels [38].

Although much remains to be studied about the mystery of the mechanism of chlorotoxin or chlorotoxin-like peptides, we have also confirmed that chlorotoxin inhibits the activity of hMMP2 [20]. Very recently, a toxin BmKCL1 has also been identified as a chlorotoxin-like peptide (68% identity), which inhibits tumour metastasis by inhibiting the current of glioma-specific chloride channel (GCC) and at least two other toxin-binding proteins (35 kDa and 80 kDa) inhibiting the proliferation of glioma cell [39,40]. Studies by Maertens and co-workers [36] indicate that the chlorotoxin does not affect the current induced by the volume-regulated (VRCC), calcium-activated (CaCC), glioma-specific chloride channel (GCC) and the CFTR channel. In this situation, the actual identity and mechanism through which ion channels participate in the antitumor activity of chlorotoxin are elusive; however, the protein that binds with chlorotoxin extracellularly has been established as MMP2 [37]. Furthermore, another chlorotoxin-like peptide, *i.e.*, GaTx1, which has 75% sequence identity with chlorotoxin, has been isolated and characterized by Fuller and co-workers [41]. Interestingly, GaTx1 selectively inhibits the CFTR channel only when applied from a cytoplasmic site in an ATP-dependant manner, whereas GaTx1 does not show any activity on CLC1, CLC2, CLC3, CaCC and the ligand-gated chloride channel formed by the GABAc receptor, when applied to the extracellular side at a concentration normally inhibiting the CFTR channel (IC_50_ = 48 nM in the closed state). It is surprising to note that despite the high level of sequence identity (75%), there is no activity on these targets. It has also been reported that chlorotoxin does not inhibit the CFTR channel, when applied from an extracellular or a cytoplasmic site [36,41]. Thus, there is an urgent need for the identification of more chlorotoxin and/or chlorotoxin-like peptides, so that these orphan toxins can be arranged in functionally-identical classes, like many other VGKC and VGSC blocker families [2,3,6]. 

Gliomas with their highly invasive potential are derived from glial support cells in the brain and mostly are of astrocytic origin. The penetrating or spreading ability of even low-grade gliomas precludes their successful therapy even in this era of advanced surgical and radiological techniques. At least 36,000 primary brain tumours are reported each year in the USA alone [40]. The molecular mechanisms of brain tumour invasion are complex and challenging. It includes modification of receptors, degradation and remodelling of extra-cellular matrix by tumour-secreted metalloproteases. It has been well established by *in vitro* and *in vivo* glioma models that extracellular matrix components deposit at the confrontation zone between normal and glioma brain tissues [37]. MMP2 activity, as well as upregulation have been observed not only in brain tumours, but also in breast, colon, skin, lung, prostate and ovarian cancer [42]. Research efforts aimed at exploring alternative therapies against gliomas led to the discovery of chlorotoxin. Chlorotoxin selectively binds gliomas and other tumours of neuroectodermal origin via a surface-bound complex, which includes MMP2 [43]. Chlorotoxin turned the focus of scientist towards its use as a possible template for drug design. In fact, chlorotoxin-derived drug TM601, which is in a clinical phase III trial, binds specifically with high affinity to tumour cells of the brain and not the normal brain tissues [17,37]. This fascinating story led us to confirm the activity of chlorotoxin and some chlorotoxin-like peptides (Bs-Tx7 and 8) from scorpion (*B. sindicus*) venom. As the amount of purified peptide is one of the challenges in toxin research, we successfully synthesized these toxins using two strategies of cysteine modification: (1) cysteine protected with an acetamidomethyl (Acm) group; and (2) cysteine protected with trityl (trt) residue. The oxidation of reduced peptides after the removal of protecting groups was monitored at 4 °C, as well as at room temperature (20–25 °C). We successfully oxidized 20%–30% peptide using the second strategy at room temperature, and all of the synthesized peptides were fully active, indicating complete or near complete folding to the native-like conformation. Chlorotoxin, a positive control for the human MMP2 assay, was also synthesized using the same strategy. The IC_50_ of the synthetic chlorotoxin found using synthetic FRET substrate was 0.09 µM as compared to 625 µM determined by zymography. The data show that the FRET substrate assay is approximately 7000-times more sensitive than zymography [20].

## 4. Experimental Section

### 4.1. Isolation, Purification and Characterization

Scorpion *Buthus sindicus* venom was collected and processed as described earlier [13,18,32]. The venom protein and peptide components were preliminary fractionated in a single step using RP-HPLC on a Nucleosil 7C18 column (100 × 4.6 mm; Macherey-Nagel, Düren, Germany) and a 0.1% TFA-acetonitrile system (Figure S1; [18]).

Final purification of the synthetic peptides, as well as re-chromatography of scorpion toxin “Bs-Tx7” was achieved on C2/C18 column (µRP C2/C18, 3-µm particle size, 100 × 4.6 mm, Amersham Biosciences, Buckinghamshire, UK), as recently described by us [20,21]. The eluate was monitored at 214 nm. The peaks were manually collected, vacuum-dried and subjected to polyacrylamide gel electrophoresis or mass spectrometry. Gelatin zymography was performed according to the method described by Heussen and Dowdle [44] to determine protease activity, and inhibition was detected in terms of the intensity of the clear band of catalysis against a blue background. Human MMP2 (Sigma-Aldrich, Deisenhofen, Germany) as used as the standard protease for testing the inhibitory activity of chlorotoxin and Bs-Tx7 [20].

For purity and mass analysis, 0.5 µL of purified Bs-Tx7 mixed with 0.5 µL DHB-matrix (10 mg/mL (*w/v*) 2,5-dihydroxybenzoic acid in 60% ethanol containing 0.1% (*v/v*) TFA) were applied on a polished steel target plate for MALDI-TOF-MS [45,46]. The sample was allowed to dry, and signals were generated by shining 50–100 laser shots on the sample using reflector positive mode and analysed between 3000 and 6000 Da (Reflex IV, Bruker Daltonics, Bremen, Germany). Calibration was performed using a peptide calibration standard from Bruker. Raw data were analysed using the software Flex Analysis 2.4 [20,21].

### 4.2. Primary Structure Elucidation of Bs-Tx7

Purified Bs-Tx7 was oxidized using hydrogen peroxide and formic acid (1:9, *v/v*) according to the method described by Sanger [47]. Briefly, Bs-Tx7 (50 μg, oxidized), dissolved in 200 μL of 50 mM ammonium bicarbonate buffer pH 8.3 containing 10% acetonitrile, was digested with 10 µL trypsin (0.1 mg/mL) for 18 h at 37 °C [48]. The digested peptides were fractionated by RP-HPLC on a C12 Protein and Peptide column (250 × 4.6 mm, Jupiter 4 µ Proteo90A; Phenomenox, Aschaffenburg, Germany) using a 0.1% TFA (Buffer A) and acetonitrile (Buffer B) gradient system (*i.e.*, 0%–40% B in 50 min) at a flow rate of 1 mL/min. 

Molecular mass measurements of the resulting tryptic peptides were performed using the electrospray ionization technique in positive ion mode [49] using Quadruple-TOF MS (QSTAR-XL, Applied Biosystems, Foster City, CA, USA). The data were acquired using Analyst 1.4.1 software (Applied Biosystems, Foster City, CA, USA) over a mass range of 600–2000 amu (atomic mass units). The molecular mass of proteins/peptides was generated from several multiply-charged peaks using the Bayesian Protein Reconstruct option in Bioanalyst 1.4 software (Applied Biosystems, Foster City, CA, USA). The instrument was calibrated using apomyoglobin (16,951.3 Da) and rennin substrate (1757.93 Da).

Amino acid sequence analysis of the intact peptide (Bs-Tx7), as well as fragments obtained after enzymatic digestion was performed with automatic Edman degradation in a gas phase sequencer (Procise 491, Applied Biosystems, Foster City, CA, USA) equipped with an on-line PTH (phenylthiohydantoin)-analyzer (785A, Perkin–Elmer, Waltham, MA, USA) for the detection of phenylthiohydantoin (PTH) derivatives [50,51]. Approximately 20–50 pM of purified samples were applied on the cartridge filter previously treated with polybrene. Cysteines were determined either as cysteic acid (after performic acid oxidation) or as PTH-DTT-dehydro-Ser. The repetitive yields during sequencing of these proteins and peptides were >95%. In order to further complement the established complete primary structure of Bs-Tx7, shotgun analysis of the whole crude scorpion (*B. sindicus*) venom was also performed as recently described by us [19] from where the complete sequence of the Bs-Tx7 was successfully curetted. 

### 4.3. Solid Phase Peptide Synthesis of Bs-Tx7

Scorpion toxin Bs-Tx7 and Bs-Tx8, as well as a known standard toxin (*i.e.*, chlorotoxin) and human matrix metalloprotease 2 (hMMP2) FRET peptidyl substrate were synthesized by a solid-phase technique using the *N*-(9-fluorenyl) methoxycarbonyl (Fmoc) strategy on a Syro-II synthesizer (MultiSyn Tech, Witten, Germany), purified (RP-HPLC), oxidized and analysed (RP-HPLC, MALDI TOF MS, CD), as recently described by us [20,21,22].

### 4.4. Continuous Fluorescence MMP2 Assay 

The continuous fluorescence enzyme assay was performed as described by Lützner and co-workers [52] with slight modifications by us [20]. Briefly, the effect of Bs-Tx7 was determined by activating hMMP2 in the absence (control) and presence (test) of different concentrations of the toxin. The substrate at a final concentration of 20 µM in a 200-µL reaction mixture was then processed with control and test hMMP2 (1.8 fM in the reaction mixture). Formation of the fluorescent product was recorded over 2 h, using the same conditions as described above. The data obtained were analysed on SoftMax^®^ Pro 5 software (Molecular Devices, Sunnyvale, CA, USA).

For confirmation of the presence of a scissile peptide bond (*i.e.*, Gly-Ile) for hMMP2 in the Bs-Tx7 sequence, both reduced and oxidized Bs-Tx7 were subjected to hMMP2 catalysis under the aforementioned identical assay conditions. After incubation time (*i.e.*, 2 h at 37 °C), total reaction-mix samples were subjected to C2/C18 RP-HPLC separation, as described in Section 4.2. Untreated native Bs-Tx7 was used as the control. The peaks were manually collected, vacuum-dried and subjected to Edman sequencing for identification. 

### 4.5. In Silico Studies 

The known three-dimensional structural coordinates of the chlorotoxin and chlorotoxin-like peptides for the search of the best template, superimposition and structural evaluation were retrieved from the Brookhaven Protein Data Bank [28], except the toxin Lqh8/6 [53], whose structural coordinates were kindly provided by Dr. Constantin T. Craescu (Orsay, France). All known toxin sequences were obtained from Swiss Protein Data Bank [23]. Similarities between different sequences were analysed using the program FASTA [24] and/or BLAST [25]. Multiple sequence alignment was carried out using the program CLUSTAL X [26] with default parameters and manually adjusted where necessary. The phylogenetic lineage was calculated using the phylogeny inference program Phylip [27].

Three-dimensional structural models of Bs-Tx7 and GaTx1 were constructed using the known crystal/NMR structural templates. Homology models were generated automatically using the software MODELLER 8.0 [29,30] and/or the online Swiss-Modeling Server [54]. The predicted models have been evaluated for geometry, stereochemistry and energy distributions. The ENERGY command of the MODELLER and/or PROSA has been used for determining reliability. The models have been further evaluated by the programs “PROCHECK” and “WHATCHECK” [55,56]. Variability among the models has been compared by superposition of Cα traces and the backbones onto the template structure and calculated as RMSD. WEBLAB Viewer Pro (v4.0) (MSI, San Diego, CA, USA)was used for visualization of the structures.

## 5. Conclusions

The work described here and in previous reports provides a molecular basis for the diversity of the chlorotoxin family of peptides from scorpion venoms. The newly-isolated peptide ‘Bs-Tx7’ from scorpion *Buthus sindicus* venom along with its closest analogue “GaTx1” from *L. quinquestriatus hebraeus* venom certainly constitute a subfamily of peptides targeting the CFTR channel, in contrast to chlorotoxin and chlorotoxin-like peptides, which bind to the MMP2/GCC/annexin-A2 complex. The presence of a scissile peptide bond (*i.e.*, Gly-Ile) for hMMP2, whose activity is increased in the case of tumour malignancy, nicely provides the molecular basis for this differentiation. The effect of hMMP2 on Bs-Tx7 or *vice versa*, observed using the FRET peptidyl substrate, showed approximately a 60% increase in the activity of hMMP2, thus also indicating the importance of this toxin (as the first natural substrate) in diseases associated with decreased MMP2 activity [57]. Although we have not performed CFTR channel inhibition activities, the lack of hMMP2 inhibition and very high sequence homology (82%) with GaTx1 suggest that the CFTR channel is probably the cellular target of Bs-Tx7. However, more studies are needed to investigate the CFTR channel-modulating activities of Bs-Tx7 in comparison with GaTx1.

## Supplementary Materials

The following are available online at www.mdpi.com/2072-6651/8/2/36/s1.

## Abbreviations

BBB, blood brain barrier; BmK, *Buthus martensii* Karsch; Bom, *Buthus occitanus mardochei*; Bot, *Buthus occitanus tunetanus*; Bs, *Buthus sindicus*; Cltx/CHL, chlorotoxin; CD, circular dichroism; CFTR, cystic fibrosis transmembrane conductance regulator; ESI, electrospray ionization; FRET, fluorescence resonance energy transfer; GaTx1, peptidyl inhibitor of CFTR channel from scorpion venom; GCC, glioma-specific chloride channel or modulating receptor; SCNs, short-chain neurotoxins; Lqh, *Leiurus quinquestriatus hebraeus*; Lqq, *Leiurus quinquestriatus quinquestriatus*; Os, *Orthochirus scrobiculosus*; MALDI TOF, matrix-assisted laser desorption time of flight; hMMP, human matrix metalloproteinase; MS, mass spectrometry; RMSD, root mean squared deviation; Tx, toxin; VGC, voltage-gated chloride channel.

## References

[1] Chippaux J.P., Goyffon M. (2008). Epidemiology of scorpionism: A global appraisal. Acta Tropica.

[2] Possani L.D., Becerril B., Delepierre M., Tytgat J. (1999). Scorpion toxins specific for Na^+^-channels. Eur. J. Biochem..

[3] Possani L.D., Merino E., Corona M., Bolivar F., Becerril B. (2000). Peptides and genes coding for scorpion toxins that affect ion-channels. Biochimie.

[4] Fry B.G., Roelants K., Champagne D.E., Scheib H., Tyndall J.D., King G.F., Nevalainen T.J., Norman J.A., Lewis R.J., Norton R.S. (2009). The toxicogenomic multiverse: Convergent recruitment of proteins into animal venoms. Annu. Rev. Genomics Hum. Genet..

[5] Goudet C., Chi C.W., Tytgat J. (2002). An overview of toxins and genes from the venom of the Asian scorpion *Buthus martensi* Karsch. Toxicon.

[6] Tytgat J., Chandy K.G., Garcia M.L., Gutman G.A., Martin-Eauclaire M.-F., van der Walt J.J., Possani L.D. (1999). A unified nomenclature for short-chain peptides isolated from scorpion venoms: α-KTx molecular subfamilies. Trends Pharmacol. Sci..

[7] Valdivia H.H., Kirby M.S., Lederer W.J., Coronado R. (1992). Scorpion toxins targeted against the sarcoplasmic reticulum Ca^+2^-release channel of skeletal and cardiac muscle. Proc. Natl. Acad. Sci. USA.

[8] Martin-Eauclaire M.F., Ceard B., Bosmans F., Rosso J.P., Tytgat J., Bougis P.E. (2005). New Birtoxin analogs from *Androctonus australis* venom. Biochem. Biophys. Res. Commun..

[9] Zhu S., Gao B. (2006). Molecular characterization of a new scorpion venom lipolysis activating peptide: Evidence for disulfide bridge-mediated functional switch of peptides. FEBS Lett..

[10] Gilles N., Blanchet C., Shichor I., Zaninetti M., Lotan I., Bertrand D., Gordon D. (1999). A scorpion alpha-like toxin that is active on insects and mammals reveals an unexpected specificity and distribution of sodium channel subtypes in rat brain neurons. J. Neurosci..

[11] DeBin J.A., Maggio J.E., Strichartz G.R. (1993). Purification and characterization of chlorotoxin, a chloride channel ligand from the venom of the scorpion. Am. J. Physiol..

[12] Rosso J.P., Rochat H. (1985). Characterization of ten proteins from the venom of the Moroccan scorpion *Androctonus mauretanicus mauretanicus*, six of which are toxic to the mouse. Toxicon.

[13] Ali S.A., Stoeva S., Schütz J., Kayed R., Abbasi A., Zaidi Z.H., Voelter W. (1998). Purification and primary structure of low molecular mass peptides from scorpion (*Buthus sindicus*) venom. Comp. Biochem. Physiol. A.

[14] Dardevet L., Rani D., Aziz T.A., Bazin I., Sabatier J.M., Fadl M., Brambilla E., de Waard M. (2015). Chlorotoxin: A helpful natural scorpion peptide to diagnose glioma and fight tumor invasion. Toxins.

[15] Mamelak A.N., Rosenfeld S., Bucholz R., Raubitschek A., Nabors L.B., Fiveash J.B., Shen S., Khazaeli M.B., Colcher D., Liu A. (2006). Phase I single-dose study of intracavitary-administered iodine-131-TM-601 in adults with recurrent high-grade glioma. J. Clin. Oncol..

[16] Zhao L., Shi X., Zhao J. (2015). Chlorotoxin-conjugated nanoparticles for targeted imaging and therapy of glioma. Curr Top Med Chem..

[17] Veiseh M., Gabikian P., Bahrami S.B., Veiseh O., Zhang M., Hackman R.C., Ravanpay A.C., Stroud M.R., Kusuma Y., Hansen S.J. (2007). Tumor paint: A chlorotoxin:Cy5.5 bioconjugate for intraoperative visualization of cancer foci. Cancer Res..

[18] Ali S.A., Stoeva S., Grossmann J.G., Abbasi A., Voelter W. (2001). Purification, characterization, and primary structure of four depressant insect-selective neurotoxin analogs from scorpion (*Buthus sindicus*) venom. Arch. Biochem. Biophys..

[19] Ali S.A., Yang D., Jackson T.N.W., Undheim E.A.B., Koludarov I., Wood K., Jones A., Hodgson W.C., McCarthy S., Ruder T. (2013). Venom proteomic characterization and relative antivenom neutralization of two medically important Pakistani Elapid snakes (*Bungarus sindanus* and *Najanaja*). J Proteomics.

[20] Alam M., Ali S.A., Abbasi A., Kalbacher H., Voelter W. (2012). Design and synthesis of a peptidyl-FRET substrate for tumor marker enzyme human matrix metalloprotease-2 (hMMP-2). Int. J. Pept. Res. Ther..

[21] Ali S.A., Alam M., Abbasi A., Kalbacher H., Schaechinger T.J., Hu Y., Zhijian C., Li W., Voelter W. (2014). Structure–activity relationship of a highly selective peptidyl inhibitor of Kv1.3 voltage-gated K^+^-channel from Scorpion (*B. sindicus*) venom. Int. J. Pept. Res. Ther..

[22] Kohl B., Rothenberg I., Ali S.A., Alam M., Seebohm G., Kalbacher H., Voelter W., Stoll R. (2015). Solid phase synthesis, NMR structure determination of α-KTx3.8, its *in silico* docking to Kv1.x potassium channels, and electrophysiological analysis provide insights into toxin-channel selectivity. Toxicon.

[23] Bairoch A., Boeckmann B. (1991). The SWISS-PROT protein sequence data bank. Nucleic Acids Res..

[24] Pearson W.R. (1995). Comparison of methods for searching protein sequence databases. Protein Sci..

[25] Altschul S.F., Madden T.L., Schäffer A.A., Zhang J., Zhang Z., Miller W., Lipman D.J. (1997). Gapped BLAST and PSI-BLAST: A new generation of protein database search programs. Nucleic Acids Res..

[26] Thompson J.D., Gibson T.J., Plewniak F., Jeanmougin F., Higgins D.G. (1997). The Clustal X windows interface: Flexible strategies for multiple sequence alignment aided by quality analysis tools. Nucleic Acids Res..

[27] Felsenstein J. (1993). PHYLIP: Phylogeny Inference Package.

[28] Berman H.M., Westbrook J., Feng Z., Gilliland G., Bhat T.N., Weissig H., Shindyalov I.N., Bourne P.E. (2000). The Protein Data Bank. Nucleic Acids Res..

[29] Šali A., Blundell T.L. (1990). Definition of general topological equivalence in protein structures: A procedure involving comparison of properties and relationships through simulated annealing and dynamic programming. J. Mol. Biol..

[30] Šali A., Blundell T.L. (1993). Comparative protein modelling by satisfaction of spatial restraints. J. Mol. Biol..

[31] Santibáñez-López C.E., Francke O.F., Ureta C., Possani L.D. (2016). Scorpions from Mexico: From species diversity to venom complexity. Toxins.

[32] Ali S.A., Wang B., Alam M., Beck A., Stoeva S., Voelter W., Abbasi A., Duszenko M. (2006). Structure-activity relationship of an α-toxin Bs-Tx28 from scorpion (*Buthus sindicus*) venom suggests a new α-toxin subfamily. Arch. Biochem. Biophys..

[33] Pereira A.M., Strasberg-Rieber M., Rieber M. (2005). Invasion-associated MMP-2 and MMP-9 are up-regulated intracellularly in concert with apoptosis linked to melanoma cell detachment. Clin. Exp. Metastasis.

[34] Jentsch T.J., Günther W. (2005). Chloride channels: An emerging molecular picture. BioEssays.

[35] Soroceanu L., Gillespie Y., Khazaeli M.B., Sontheimer H. (1998). Use of chlorotoxin for targeting of primary brain tumors. Cancer Res..

[36] Maertens C., Wei L., Tytgat J.G., Droogmans B., Nilius B. (2000). Chlorotoxin does not inhibit volume-regulated, calcium-activated and cyclic AMP-activated chloride channels. Br. J. Pharmacol..

[37] Deshane J., Garner C.C., Sontheimer H. (2003). Chlorotoxin inhibits glioma cell invasion via matrix metalloproteinase-2. J. Biol. Chem..

[38] McFerrin M.B., Sontheimer H. (2006). A role for ion channels in glioma cell invasion. Neuron Glia. Biol..

[39] Fan S., Sun Z., Jiang D., Dai C., Ma Y., Zhao Z., Liu H., Wu Y., Cao Z., Li W. (2010). BmKCT toxin inhibits glioma proliferation and tumor metastasis. Cancer Lett..

[40] Fu Y.J., Yin L.T., Liang A.H., Zhang C.F., Wang W., Chai B.F., Yang J.Y., Fan X.J. (2007). Therapeutic potential of chlorotoxin-like neurotoxin from the Chinese scorpion for human gliomas. Neurosci Lett..

[41] Fuller M.D., Thompson C.H., Zhang Z.R., Freeman C.S., Schay E., Szakács G., Szakács G., Bakos E., Sarkadi B., McMaster D. (2007). State-dependent inhibition of cystic fibrosis transmembrane conductance regulator chloride channels by a novel peptide toxin. J. Biol. Chem..

[42] Veiseh O., Sun C., Fang C., Bhattarai N., Gunn J., Kievit F., Du K., Pullar B., Lee D., Ellenbogen R.G. (2009). Specific targeting of brain tumors with an optical/magnetic resonance imaging nanoprobe across the blood-brain barrier. Cancer Res..

[43] Veiseh O., Gunn J.W., Kievit F.M., Sun C., Fang C., Lee J.S., Zhang M. (2009). Inhibition of tumor-cell invasion with chlorotoxin-bound super paramagnetic nanoparticles. Small.

[44] Heussen C., Dowdle E.B. (1980). Electrophoretic analysis of plasminogen activators in polyacrylamide gels containing sodium dodecyl sulfate and copolymerized substrates. Anal. Biochem..

[45] Tanaka K., Waki H., Ido Y., Akita S., Yoshida Y., Yoshida T. (1988). Protein and polymer analyses up to *m/z* 100,000 by laser ionization time-of flight mass spectrometry. Rapid Commun Mass Spectrom..

[46] Strupat K., Karas M., Hillenkamp F. (1991). 2,5-Dihidroxybenzoic acid: A new matrix for laser desorption-ionization mass spectrometry. Int. J. Mass Spectrom. Ion Processes.

[47] Sanger F. (1949). Fractionation of oxidized insulin. Biochem. J..

[48] Hirs C.H.W. (1967). Enzyme Structure, Methods in Enzymology.

[49] Fenn J.B., Mann M., Meng C.K., Wong S.F. (1990). Electrospray ionization—Principles and practice. Mass Spectrom. Rev..

[50] Hewick R.M., Hunkapillar M.W., Hood L.E., Dreyer W.J. (1981). A gas-liquid solid phase peptide and protein sequenator. J. Biol. Chem..

[51] Edman P., Begg G. (1967). A protein sequenator. Eur. J. Biochem..

[52] Lützner N., Pätzold B., Zoll S., Stehle T., Kalbacher H. (2009). Development of a novel fluorescent substrate for Autolysin E, a bacterial type II amidase. Biochem. Biophys. Res. Commun..

[53] Adjadj E., Naudat V., Quiniou E., Wouters D., Sautiére P., Craescu C.T. (1997). Solution structure of Lqh-8:6, a toxin-like peptide from scorpion venom. Eur. J. Biochem..

[54] Swiss-Modeling Server. http://swissmodel.expasy.org.

[55] Laskowski R.A., McArthur M.W., Moss D.S., Thornton J.M. (1993). PROCHECK—A program to check the stereochemical quality of protein structures. J. Appl. Crystallogr..

[56] Vriend G. (1990). WHAT IF: A molecular modeling and drug design program. J. Mol. Graph..

[57] Lim N.K., Villemagne V.L., Soon C.P., Laughton K.M., Rowe C.C., McLean C.A., Masters C.L., Evin G., Li Q.X. (2011). Investigation of matrix metalloproteinases, MMP-2 and MMP-9, in plasma reveals a decrease of MMP-2 in Alzheimer's disease. J Alzheimers Dis..

