# Supplementary material for: Structure-Activity Relationship of Chlorotoxin-Like Peptides
Source: Toxins (Basel). 2016 Feb 2;8(2):36. doi: 10.3390/toxins8020036 (PMC4773789; doi:10.3390/toxins8020036)
Supplement: Supplementary file 1 [file toxins-08-00036-s001.pdf]

Supplementary Materials: Structure-Activity Relationship of Chlorotoxin-Like Peptides

Syed Abid Ali, Mehtab Alam, Atiya Abbasi, Eivind A. B. Undheim, Bryan Grieg Fry, Hubert Kalbacher and Wolfgang Voelter

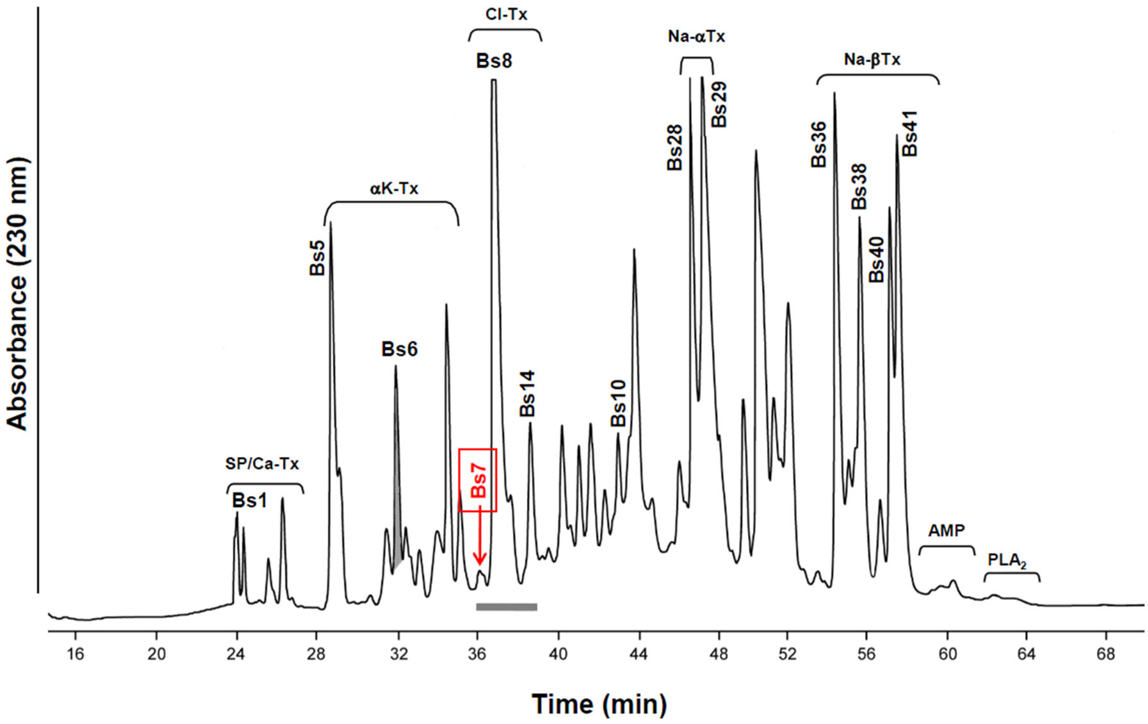

Figure S1. Single-step reversed phase high performance liquid chromatography of scorpion (*B. sindicus*) venom (~3 mg) on a Nucleosil 7C18 column (250 × 10 mm; Macherey-Nagel, Düren, Germany). The following conditions for RP-HPLC separation were used: Eluent A, 0.1% trifluoroacetic acid in water; Eluent B, 100% acetonitrile containing 0.05% TFA; gradient program, 15% B for 5 min, followed by 70% B for 90 min at a flow rate of 1 mL/min [18]. The UV absorbance of the eluate was monitored at 230 nm. Identification and annotation of the major peaks were based on mass spectrometry and our previously-published results [13,18,21,33].

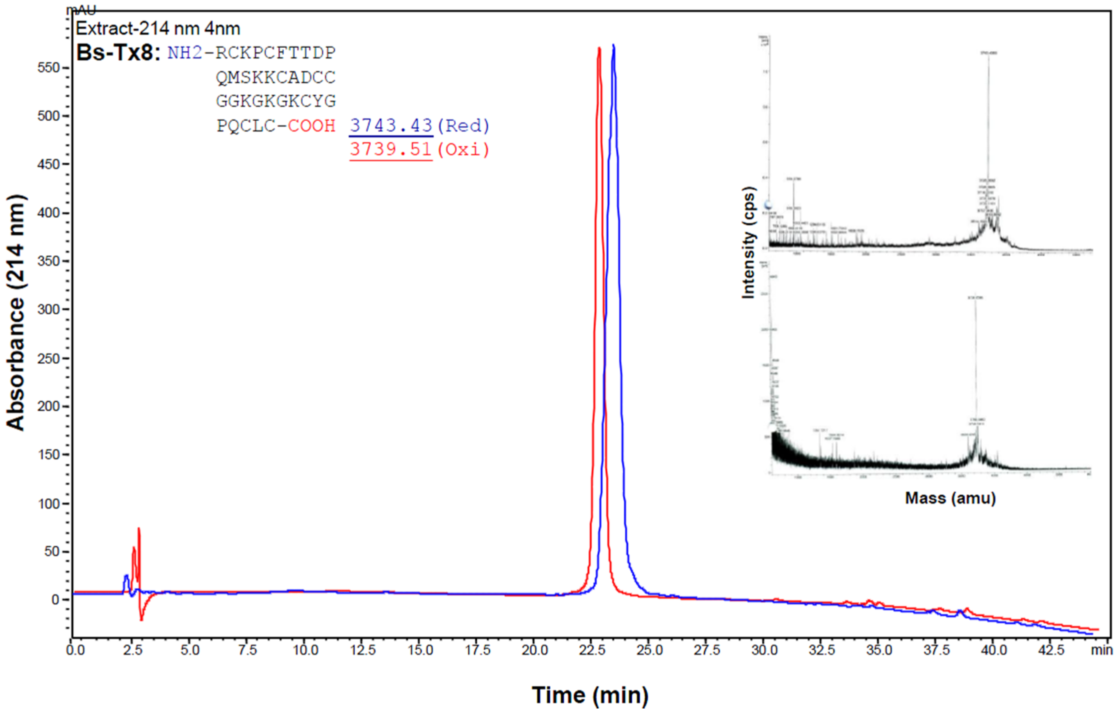

Figure S2. Solid-phase peptide synthesis of Bs-Tx8 ([13]; a close homologue of chlorotoxin) using a Syro-II synthesizer and oxidized as described by us [19,33,43]. Superimposed are the chromatograms of the purified synthetic Bs-Tx8 in reduced (blue) and oxidized (red) forms. The sample was loaded on an RP-HPLC column (μRP-C2/C18) and manually collected using a gradient program as described in Figure 1a. Insert: the average molecular masses of the collected peptides were determined by MALDI-TOF MS.

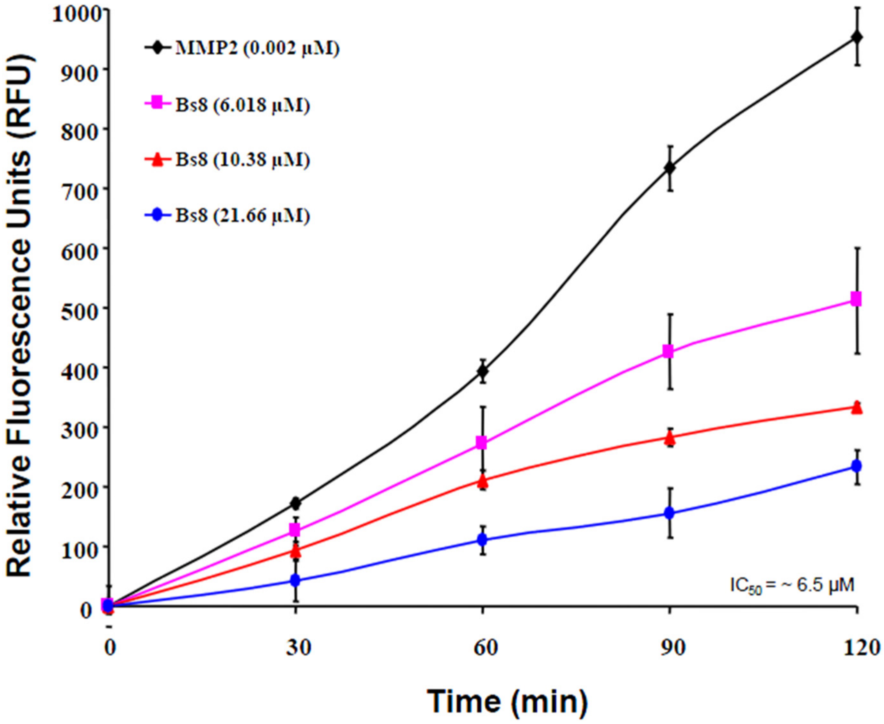

Figure S3. Time progress curves of hMMP2 showing the hydrolysis of the internally-quenched synthetic fluorescent substrate without (control) and with different concentrations of synthetic Bs-Tx8 (test). Experimental data represent the mean ± SE of three experiments.
